# Qiju Dihuang Decoction for Hypertension: A Systematic Review and Meta-Analysis

**DOI:** 10.1155/2020/9403092

**Published:** 2020-07-31

**Authors:** Shuo Zhang, Xue Bai, Zhen-Lin Chen, Jia-Jia Li, Yan-Yan Chen, Yu-Ping Tang

**Affiliations:** ^1^School of Traditional Chinese Medicine, Beijing University of Chinese Medicine, Beijing 100029, China; ^2^Key Laboratory of Shaanxi Administration of Traditional Chinese Medicine for TCM Compatibility, Shaanxi University of Chinese Medicine, Xi'an 712046, Shaanxi, China; ^3^Graduate School, Shaanxi University of Chinese Medicine, Xi'an 712046, Shaanxi, China

## Abstract

**Objective:**

To systematically evaluate the efficacy of Chinese herbal medicine Qiju Dihuang Decoction (QDD) for hypertension.

**Methods:**

A comprehensive literature search of randomized controlled trials using QDD to treat hypertension was conducted in 7 electronic databases, including Chinese databases. Subjects and abstracts of the trials were read in NoteExpress for preliminary screening, and the full text was read for further screening. The data extraction table was made for the selected 19 trials, and risk of bias was assessed by using the Cochrane collaboration tool, followed by data analysis using Rev Man 5.3.

**Results:**

The antihypertensive efficacy of QDD is 1.45 times that of antihypertensive drugs and 1.56 times that of conventional therapies, which can also reduce the endothelin level. QPAD exhibits an antihypertensive effect, and its clinical efficacy is 1.34 times and 1.61 times that of antihypertensive drugs, which can not only significantly lower the diastolic blood pressure but also reduce the 24 h mean ambulate blood pressure. At the same time, it can decrease the TCM syndrome score, inhibit the inflammation, protect the renal function, reduce the insulin resistance, and improve the life quality of patients.

**Conclusion:**

QDD can effectively reduce blood pressure and improve the life quality of patients with hypertension, which plays a certain role in preventing hypertension complications. However, due to the methodological deficiencies, more rigorous randomized controlled trials will be needed in the future to provide stronger evidence.

## 1. Introduction

Hypertension is a common clinical cardiovascular disease with systemic arterial pressure increase as the main sign, which not only causes headache, dizziness, and other clinical symptoms but also damages important organs such as the heart, brain, kidney and eyes, and it further produces multiple system lesions or dysfunction [1]. Its high morbidity and mortality bring physical and mental suffering to patients [2]. In recent years, hypertension has become a global public health problem involving over one billion people worldwide [3, 4]. Patients with hypertension require a long-term medication with diuretics, angiotensin-converting enzyme inhibitors, and calcium antagonists that are widely used. However, they frequently cause some contraindications and adverse reactions, such as irritant dry cough, hyperkalemia, sexual dysfunction, and renal deterioration, which seriously affects the life quality of many patients. And after using these drugs, about half of patients cannot effectively control their blood pressure [5]. Traditional Chinese medicine (TCM) is often used to treat patients with hypertension [6, 7], which can not only achieve good clinical efficacy but also may reduce the adverse reactions. Moreover, there are also certain advantages that TCM is applied for treating the complications of hypertension. Hypertension belongs to vertigo or headache in TCM. The first treatises on “vertigo” and “headache” appeared in *Inner Canon of Yellow Emperor* (Huang Di Nei Jing in Chinese). TCM believes that the onset of hypertension is mainly due to wind, phlegm, deficiency, blood stasis, and other diseases caused by the wind dizzy internal movement, phlegm and blood stasis, and Qi and blood block or Qing-qiao loss of nourishing, which results in the imbalance between Qi and blood and Yin and Yang. Therefore, the nature of hypertension is usually described as deficiency in origin and excess in superficiality.

Qiju Dihuang Decoction (QDD), recorded in the Qing Dynasty *Measles Complete Book* (Ma Zhen Quan Shu in Chinese), is one of the most commonly used TCM formulae for the syndrome of “liver and kidney Yin deficiency,” mainly used for vertigo, tinnitus, eyes acerbity photophobia, and vision dim [8, 9]. QDD is composed by 8 kinds of herbs, including Lycii Fructus (Gouqizi, fruit from *Lycium barbarum* L.), Chrysanthemi Flos (Juhua, inflorescence from *Chrysanthemum morifolium* Ramat.), Rehmanniae Radix Praeparata (Shudihuang, root tuber from *Rehmannia glutinosa* Libosch.), Dioscoreae Rhizoma (Shanyao, rhizome from *Dioscorea opposita* Thunb.), Corni Fructus (Shanzhuyu, pulp from *Cornus officinalis* Sieb. et Zucc.), Moutan Cortex (Mudanpi, velamen from *Paeonia suffruticosa* Andr.), Poria (Fuling, sclerotium from *Poria cocos* Schw. Wolf), and Alismatis Rhizoma (Zexie, tuber from *Alisma orientale* Sam. Juzep.). Gouqizi and Shudihuang can benefit the kidney Yang and nourish essence, Juhua can clear the liver and improve the vision, Shanyao can tonify the spleen and nourish the kidney, Shanzhuyu can nourish the kidney and liver, Mudanpi can clear away the liver-fire, Fuling can induce diuresis with bland drug, and Zexie can infiltrate to remove dampness and turbidity. Pharmacological studies have found that QDD can improve the liver fat metabolism, reduce the capillary permeability, enhance the immunity, diminish the inflammation, delay the senility, and decrease the alloxan caused by high blood glucose. QDD contains many chemical components such as cycloene terpenes, flavones, cholines, and vitamin [10, 11]. As an effective drug, QDD is widely used for the treatment of hypertension and related complications by combining with other Chinese herbal medicines. Some animal experiments proved that QDD could not only reduce blood pressure and blood lipids [12] but also lowers the ratio of middle level of renal arteriolus and lumen diameter in spontaneously hypertensive rats [13], so as to regulate renal vascular remodeling and to improve renal blood flow [14], which plays a protective role in the kidney. At the same time, by improving the ultrastructure of endothelial cells, it can regulate the mitochondrial membrane potential and calcium ion concentration of cells, affect the proliferation of endothelial cells [15], regulate the NO-NOS system, and reduce the damage of vascular endothelial cells [16]. Although there are some systematic reviews and meta-analyses on Chinese herbal medicines for the treatment of hypertension, most of them only reported the antihypertensive effect of Chinese herbal medicines and the changes in blood pressure before and after treatment and did not touch upon the life quality of patients and the prevention of complications. So far, only some studies have shown that QDD can be used alone or in combination with other TCM formulae to treat hypertension and other related diseases, but the clinical indicators and antihypertensive effects in these studies are different. In addition, the efficacy of QDD in the treatment of hypertension has not been systematically assessed. Therefore, this study was designed to evaluate the effect of QDD on hypertension patients' blood pressure, life quality, renal function, and insulin resistance as well as the potential mechanism of lowering blood pressure.

## 2. Data and Methods

This study was conducted following the Preferred Reporting Items for Systematic Reviews and Meta-analyses (PRISMA) [17].

### 2.1. Literature Search

Randomized controlled trials (RCTs) that evaluated the effect of QDD for hypertension were searched in the following 7 electronic databases: the Chinese National Knowledge Infrastructure (CNKI, from 1980 to 2020), Chinese Biomedical Literature Database (CBM, from 1978 to 2020), Wanfang Database (from 1998 to 2020), Weipu Database (from 1989 to 2020), PubMed (from 1959 to 2020), and Web of Science (from 1986 to 2020) and Springerlink (from 1996 to 2020). We used the following terms “blood pressure” OR “high blood pressure” OR “hypertension” OR “essential hypertension” OR “primary hypertension” OR “*gao xue ya*” OR “*xue ya*” AND (“Qiju Dihuang decoction” OR “Qijudihuang decoction” OR “Qi ju di huang decoction” OR “Qijudihuang tang” OR “Qiju dihuang tang” OR “Qi ju di huang tang” OR “Qijudihuangtang” AND “clinical trial” OR “randomized controlled trial” OR “randomized controlled trial” OR “*lin chuang yan jiu*” OR “*lin chuang shi yan*”. No restriction on language was applied.

### 2.2. Criteria for Literature Inclusion

#### 2.2.1. Patients

Trials were considered eligible for inclusion if the diagnosis of hypertension in the patients conformed to the diagnostic criteria established by the World Health Organization or International Hypertension Federation or the guidelines for the prevention and treatment of hypertension in China. And patients were not restricted by age, gender, or nationality. Studies were excluded if (a) the diagnostic criteria of the study object were unclear; (b) the patient had secondary hypertension; (c) the patient was accompanied by other adverse states (such as depression, insomnia, and perimenopause); (d) the patient was pregnant.

#### 2.2.2. Intervention and Control Measures

The intervention measures in the treatment group should be QDD alone or in combination with other antihypertensive treatments. And, the control group should be other antihypertensive treatments or placebo, such as conventional therapy, conventional antihypertensive drugs, and lifestyle interventions (CADLI). However, trials involving qigong, TAI Chi, acupuncture, moxibustion, massage, and cupping as common interventions will be excluded.

#### 2.2.3. Outcomes

The primary outcome was defined as blood pressure, which included antihypertensive effects, systolic blood pressure (SBP), and diastolic blood pressure (DBP). The secondary outcomes included clinical effects, TCM syndrome score, life quality, 24 h ambulatory blood pressure, angiotensin II (Ang II), endothelin (ET), insulin sensitivity index (ISI), insulin (INS), mA1b, *β*_2_-microglobulin (*β*_2_-MG), *N*-acetyl-*β*-glucosidase (NAG), high sensitivity C-reactive protein (HsCRP), and adverse reactions.

#### 2.2.4. Studies

This study included a randomized controlled trial using QDD to treat hypertension in the experimental group. Studies were excluded if (a) they were not random; (b) no control group was used; (c) the experimental design was not rigorous and statistical methods are inappropriate; (d) it was on the indeterminacy of measurement index outcome criterion; (e) they failed to obtain effective analysis data; (f) they were animal experiments; (g) they were plagiarized.

### 2.3. Data Extraction

Two researchers independently screened the titles and abstracts of potential eligible studies, and then they retrieved and reviewed the full text of the possible studies based on the inclusion and exclusion criteria and extracted the data. If there was disagreement, they agreed through discussion or submitted it to a third party for evaluation. Data extraction table mainly included the basic information of the study (author's name, title of the study, year of publication, country/region, and publication status), study characteristics (sample size, source of cases, age, gender distribution, diagnostic criteria, and inclusion and exclusion criteria), intervention and control measures (dosage form, dose, and duration), research methodology (random scheme generation, allocation hiding, blind method, incomplete result data, selective reporting, other biases, and loss of follow-up), and outcome measures.

### 2.4. Assessment of Literature Quality

According to the Cochrane collaboration tool, two reviewers independently evaluated the methodological quality of each included study. It comprised the following seven aspects: random sequence generation, allocation concealment, blind method, incomplete result data, selective reporting, and other biases [18]. The quality evaluation results of each item can be divided into three grades: “low risk,” “high risk,” and “unclear.” When necessary, the consensus on this issue was studied with the help of a third party.

### 2.5. Statistical Analysis

Statistical analysis was performed using Rev Man 5.3 software. The results of a single study were firstly described. The binomial variables were described by using relative risk (RR) and 95% confidence interval (CI), and the continuous variables were described by mean difference (MD) and 95% CI to describe the effect value of the intergroup comparison. Heterogeneity was judged on the basis of the results of *I*^2^ test. *I*^2^ > 50% indicated that the heterogeneity of inter-study was significant, and the random effect model was adopted. And, the fixed effect model was adopted when *I*^2^ < 50% [19]. Subgroup analysis was conducted by different treatment methods in the control group. Inverted funnel plots were used to determine potential publication bias when more than 10 studies were included in the meta-analysis.

## 3. Results

### 3.1. Results of Our Literature Search

**Figure 1:**
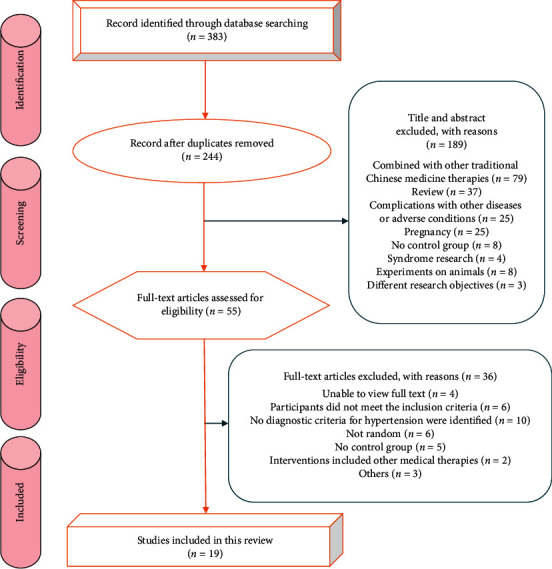
Flow diagram of literature selection.

Based on the retrieval strategy, 383 potential relevant literatures on the treatment of hypertension by QDD were preliminarily searched in 7 databases, and 244 literatures were retrieved after 139 copies were removed. After reviewing the titles and abstracts, 189 articles were excluded because they did not comply with the inclusion criteria and 55 articles initially met the intended criteria. After reading the full text, 19 eligible studies were included for meta-analysis [20–38]. The flow chart of literature retrieval and screening is shown in Figure 1.

### 3.2. Basic Characteristics of the Included Studies

Table 1 summarizes the basic characteristics of the 19 trials. All the trials were conducted in China. A total of 2043 patients with hypertension were included. Sample sizes ranged from 60 to 333. In the treatment group, QDD alone was used in 7 trials, then QDD plus antihypertensive drugs (QPAD) was used in 11 trials, and QDD plus CADLI (QPCADLI) was used in 1 trial.  Table 2 lists the detailed information of QDD ingredients used in 19 trials. For control conditions, 16 trials were treated with antihypertensive drugs, and conventional therapy was used in 2 trials; additionally, CADLI was used in 1 trial. All 19 included trials reported the efficacy of QDD in the treatment of hypertension. Antihypertensive effects and blood pressure were the primary outcomes. 14 trials reported antihypertensive effects [21–25, 27–29, 32, 34–38], and 11 reported blood pressure [20–23, 26, 29–31, 33, 37, 38]. In terms of secondary outcome indicators, clinical effects were described in 2 trials [26, 31] and TCM syndrome score was described in 1 trial [31]; furthermore, life quality was described in 2 trials [26, 31], and 24 h ambulatory blood pressure was described in 1 trial [34]. The change of Ang II was described in 2 trials [20, 36], and ET was described in 1 trial [20]; in addition, ISI and INS were described in 1 trial [30]. The changes of mA1b, *β*_2_-MG, and NAG were described in 1 trial [33], and HsCRP was described in 1 trial [36]. All included trials reported treatment duration ranging from 1 to 6 months.

### 3.3. Risk of Bias Assessment of the Literature Included in the Study

Table 3 summarizes the methodological quality of the 19 included trials. The risk of bias in the trials was assessed by using the criteria in the Cochrane Handbook for Systematic Reviews of Interventions [18]. Although randomization was announced in all of the included trials, 6 trials reported random sequence generation methods, including random number table [20, 28, 29, 32, 35] and the order of treatment [23]. However, no details were found in the domains of random allocation concealment, blinding of participants, personnel and outcome assessors, and selective outcome reporting.

### 3.4. Blood Pressure

**Figure 2:**
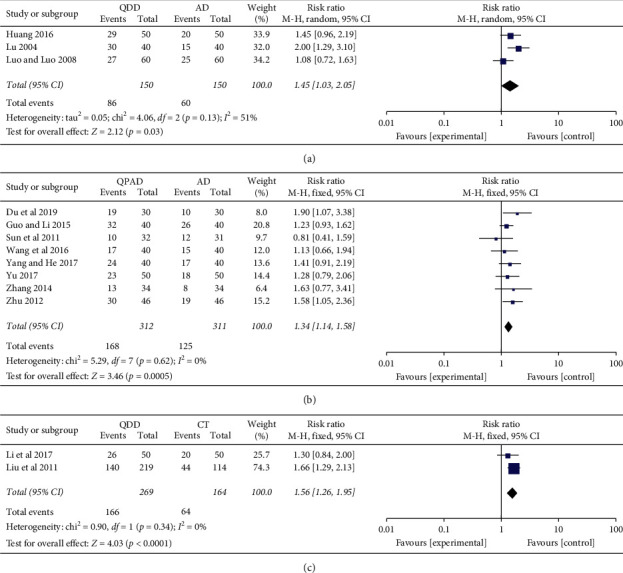
Forest plot of the trials showed antihypertensive effects in different interventions for (a) QDD vs. AD, (b) QPAD vs. AD, and (c) QDD vs. CT; abbreviations: AD: antihypertensive drugs; CT: conventional therapy; QDD: Qiju Dihuang decoction; QPAD: Qiju Dihuang decoction plus antihypertensive drugs.

The antihypertensive effect was formulated in accordance with the criteria for the diagnosis and efficacy of TCM diseases [39]. We used obvious effect as the index of antihypertensive effect, which was reported in 14 trials [21–25, 27–29, 32, 34–38]. 3 trials evaluated the antihypertensive effect of QDD compared with antihypertensive drugs, revealing that QDD antihypertensive efficiency was 1.45 times that of antihypertensive drugs (3 trials, *n* = 146; RR: 1.45; 95% CI: 1.03 to 2.05; *p*=0.03; Figure 2(a)). Meta-analysis of 8 trials comparing QPAD with antihypertensive drugs showed that QPAD antihypertensive efficiency was 1.34 times that of antihypertensive drugs (8 trials, *n* = 623; RR: 1.34; 95% CI: 1.14 to 1.58; *p*=0.0005; Figure 2(b)). QDD was compared with conventional therapy in 2 trials. According to QDD, antihypertensive efficiency was 1.56 times that of conventional therapy (2 trials, *n* = 230; RR: 1.56; 95% CI: 1.26 to 1.95; *p* < 0.0001; Figure 2(c)). However, there is no obvious difference between QPCADLI and CADLI on antihypertensive effect (1 trial, *n* = 193; RR: 1.09; 95% CI: 0.94 to 1.26; *p*=0.25).

**Figure 3:**
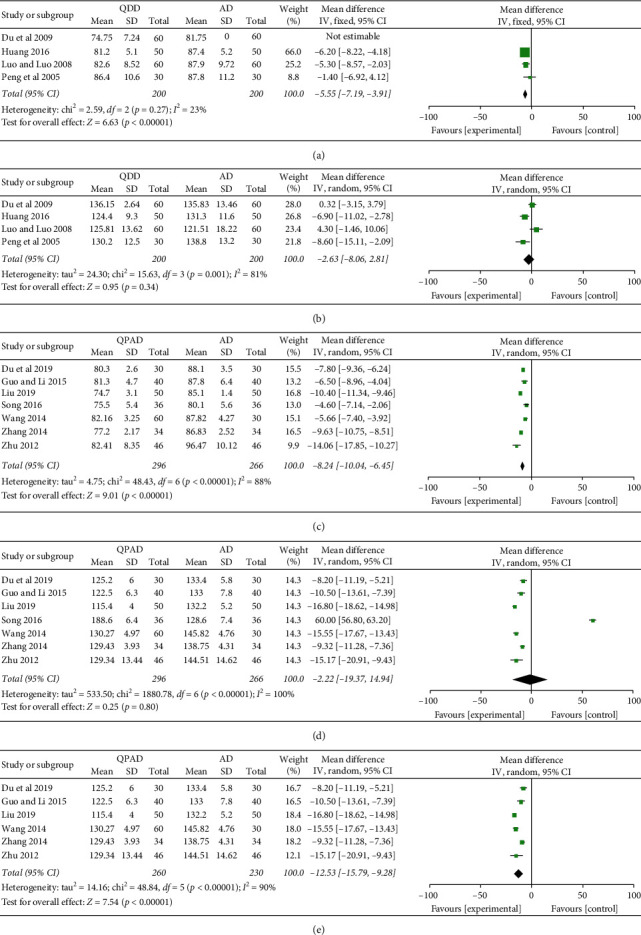
Forest plot of the trials showed blood pressure in different interventions for (a) DBP, QDD vs. AD, (b) SBP, QDD vs. AD, (c) DBP, QPAD vs. AD, (d) SBP, QPAD vs. AD, and (e) SBP, QPAD vs. AD; Abbreviations: AD: antihypertensive drugs; DBP: diastolic blood pressure; QDD: Qiju Dihuang decoction; QPAD: Qiju Dihuang decoction plus antihypertensive drugs; SBP: systolic blood pressure.

Meta-analysis of 11 trials reported the changes in blood pressure levels before and after treatment [20–23, 26, 29–31, 33, 37, 38]. 4 trials comparing QDD with antihypertensive drugs exhibited a significant lowering effect of QDD in DBP (4 trials, *n* = 400; MD: −5.55; 95% CI: −7.19 to −3.19; *p* < 0.00001; Figure 3(a)), but had no obvious difference in SBP (4 trials, *n* = 400; MD: −2.63; 95% CI: −8.06 to 2.81; *p*=0.34; Figure 3(b)). QPAD and antihypertensive drugs were compared in 7 trials, and it was found that QPAD could remarkably reduce DBP (7 trials, *n* = 562; MD: −8.24; 95% CI: −10.04 to −6.45; *p* < 0.00001; Figure 3(c)), while SBP was not significantly different from that of antihypertensive drugs (7 trials, *n* = 562; MD: −2.22; 95% CI: −19.37 to 14.94; *p*=0.80; Figure 3(d)). However, after excluding [30], combined with the results of the other 6 trials, meta-analysis identified an obvious lowering effect of QPAD in SBP (6 trials, *n* = 490; MD: −12.53; 95% CI: −15.79 to −9.28; *p* < 0.00001; Figure 3(e)).

### 3.5. Clinical Effects

**Figure 4:**
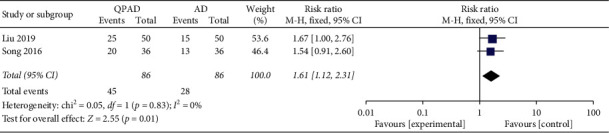
Forest plot of the trials showed clinical effects in QPAD vs. AD; Abbreviations: AD: antihypertensive drugs; QPAD: Qiju Dihuang decoction plus antihypertensive drugs.

Clinical effect was determined by the TCM syndrome score. The clinical symptoms were significantly alleviated, and the TCM syndrome score was decreased by > 70% after treatment, indicating an obvious effect. At the same time, the TCM syndrome score was decreased by 50∼70% for improvement. No improvement or aggravation of symptoms and a reduction of TCM syndrome score below 50% is invalid. Obvious effect was used as an indicator of clinical efficacy. Two trials [26, 31] described clinical effects, and meta-analysis revealed that QPAD was 1.61 times higher than antihypertensive drugs in clinical efficacy of hypertension (2 trials, *n* = 172; RR: 1.61; 95% CI: 1.12 to 2.31; *p*=0.01; Figure 4).

### 3.6. TCM Syndrome Score

The scoring method of TCM syndromes refers to the guiding principles for clinical research of new Chinese medicine [40]. According to the degree of clinical manifestations, the scores are zero, one, two, and three points respectively; moreover, the lower the score, the better the effect. Only one trial [31] reported TCM syndrome scores before and after treatment, suggesting that QPAD significantly reduced the scores in patients compared with antihypertensive drugs (1 trial, *n* = 72; MD: −3.00; 95% CI: −3.61 to −2.39; *p* < 0.00001).

### 3.7. Life Quality

SF-36 was used to evaluate the life quality of patients. And, the higher the score, the higher the life quality.

**Figure 5:**
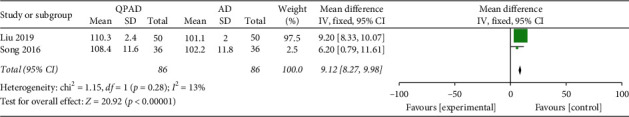
Forest plot of the trials showed life quality in QPAD vs. AD. Abbreviations: AD: antihypertensive drugs; QPAD: Qiju Dihuang decoction plus antihypertensive drugs.

2 trials reported changes in patients' life quality before and after treatment [26, 31]. Compared with antihypertensive drugs, QPAD can remarkably improve the life quality of patients (2 trials, *n* = 172; MD: 9.12; 95% CI: 8.27 to 9.98; *p* < 0.00001; Figure 5).

### 3.8. 24 h Ambulatory Blood Pressure

24 h ambulatory blood pressure was only reported in one trial [34]. Compared with antihypertensive drugs, QPAD can significantly reduce mean systolic blood pressure (1 trial, *n* = 80; MD: −13.30; 95% CI: −16.84 to −9.76; *p* < 0.00001) and diastolic blood pressure (1 trial, *n* = 80; MD: −6.25; 95% CI: −8.69 to −3.81; *p* < 0.00001) as well as the standard deviation of systolic blood pressure (1 trial, *n* = 80; MD: −2.06; 95% CI: −3.90 to −0.22; *p*=0.03). However, there is no obvious difference between QPAD and antihypertensive drugs in the standard deviation of diastolic blood pressure (1 trial, *n* = 80; MD: −0.49; 95% CI: −2.30 to 1.32; *p*=0.60).

### 3.9. Ang II and ET

Ang II at baseline and after intervention was reported by 2 trials [20, 36]. Compared with antihypertensive drugs, QPAD can significantly reduce huge quantities of Ang II levels and inhibit the activity of it (1 trial, *n* = 100; MD: −76.00; 95% CI: −98.83 to −53.17; *p* < 0.00001). However, there was no remarkable difference between QDD and antihypertensive drugs (1 trial, *n* = 72; MD: −10.86; 95% CI: −24.29 to 2.57; *p*=0.11).

Only 1 trial described ET [20], and a significant inhibition of ET bioactivity by QDD was exhibited compared with antihypertensive drugs (1 trial, *n* = 72; MD: −9.12; 95% CI: −15.18 to −3.06; *p*=0.003).

### 3.10. ISI and INS

Only one trial reported ISI at baseline and after intervention [30], and the results identified that QPAD significantly increased ISI and decreased insulin resistance (1 trial, *n* = 60; MD: 1.13; 95% CI: 0.89 to 1.37; *p* < 0.00001).

INS level was also reported by the same trial as ISI, revealing that the reduction in INS was obviously greater for QPAD than that of antihypertensive drugs alone (1 trial, *n* = 60; MD: −2.30; 95% CI: −3.23 to −1.37; *p* < 0.00001).

### 3.11. mA1b, *β*_2_-MG, and NAG

Only 1 trial reported mA1b at baseline and after intervention [33]. A remarkable reduction in mA1b with QPAD was evaluated, comparing with antihypertensive drugs (1 trial, *n* = 90; MD: −8.33; 95% CI: −14.72 to −1.94; *p*=0.01).

The effect of QPAD on *β*_2_-MG level was reported in the same trial as mA1b. Compared with antihypertensive drugs, QPAD can lower the level of *β*_2_-MG better (1 trial, *n* = 90; MD: −0.09; 95% CI: −0.13 to −0.05; *p* < 0.0001).

The same trial also reported the NAG level, in which QPAD was compared with antihypertensive drugs, and it was found to have a significant lowering effect in NAG (1 trial, *n* = 90; MD: −4.97; 95% CI: −9.92 to −0.02; *p*=0.05).

### 3.12. HsCRP Level

The HsCRP level at baseline and postintervention was reported by 1 trial that compared QPAD with antihypertensive drugs [36]. The meta-analysis exhibited an obvious lowering effect by QPAD in HsCRP (1 trial, *n* = 100; MD: −0.50; 95% CI: −0.74 to −0.26; *p* < 0.0001).

### 3.13. Adverse Reactions

No adverse reactions were reported in all trials.

### 3.14. Evaluation of Publication Bias

Since fewer than 10 trials were included in each subgroup, publication bias could not be adequately analyzed.

## 4. Discussion

TCM has certain advantages and characteristics in the treatment of hypertension. In recent years, the classic TCM formulae for treating hypertension have attracted much attention [41–43]. However, most studies only reported the antihypertensive effect but did not concern the effect of TCM on the life quality of patients and the prevention of complications. Therefore, our study aimed to systematically evaluate the efficacy of QDD on blood pressure, life quality, renal function, insulin resistance, and other aspects of patients with hypertension as well as the potential hypertensive mechanism. And, it would provide a better evidence for hypertensive patients, policymakers, and clinicians.

This systematic review and meta-analysis contained 19 trials and 2043 patients with hypertension, which evaluated the efficacy of QDD in the treatment of hypertension, including QDD vs. antihypertensive drugs, QDD vs. conventional therapy, QPAD vs. antihypertensive drugs, and QPCADLI vs. CADLI. In summary, this study has identified that QDD can significantly reduce the blood pressure, improve the antihypertensive efficiency, show an obvious lowering effect on TCM syndrome score, inhibit the inflammation, protect the kidney function, reduce the insulin resistance, and improve the life quality of patients. And, reduction of Ang II and ET levels is possibly one potential antihypertensive mechanism of QDD.

Patients with long-term hypertension will lead to a variety of pathophysiological changes, which will not only cause headache, chest tightness, palpitations, and other symptoms but also may involve various organs and cause different complications. Therefore, hypertension is a major predisposing factor for many high-risk diseases. Many studies have also showed that the treatment of hypertension can play a preventive role in the occurrence of cardiovascular and cerebrovascular diseases [44]. Effective and stable control of blood pressure can significantly reduce the incidence of coronary heart disease by around 20–25%, 35–45% lower incidence of cerebral apoplexy, and heart failure in lower rates up to 50% [45]. Compared with the current prevalence trend of hypertension, if hypertension can be effectively controlled, the number of deaths from cardiovascular and cerebrovascular diseases in adults will be decreased by 564 100 until 2030 [46]. Controlling blood pressure to target levels is the basis of preventing cardiovascular and cerebrovascular diseases in patients with hypertension, so effectively lowering blood pressure is the most critical task for treatment of hypertension. All the trials included in the meta-analysis involved measures of the antihypertensive effect of QDD. We found that both QDD alone and in combination with antihypertensive drugs can remarkably reduce diastolic blood pressure and improved the antihypertensive effect. And, the choice of QDD treatment is one of the effective ways for patients with “liver and kidney Yin deficiency” hypertension. In addition, compared with antihypertensive drugs, QPAD can significantly reduce the mean systolic blood pressure and diastolic blood pressure at 24 h as well as the standard deviation of systolic blood pressure, which suggested that the combination of QDD and antihypertensive drugs is a more optimal choice for patients who cannot effectively improve the fluctuation of blood pressure by only taking antihypertensive drugs.

This study also discovered the potential mechanism of QDD for the reduction of blood pressure and the prevention of complications. Ang II vascular remodeling is one of the important pathological changes in the pathogenesis of hypertension, such as vascular smooth muscle proliferation, apoptosis, and other lesions, which will damage vascular walls, decrease vascular compliance, and lead to the imbalance of vascular active substances and disorder hemodynamic, all of which will lead to increase blood pressure. And, it was also reported to regulate the constriction of arterioles throughout the body and increase blood pressure [47]. In addition, when vascular endothelial cells are dysfunctional, vasomotor substances (such as ET) and growth -promoting factors are released abnormally, then blood vessel will narrow and peripheral resistance will increase, which also can promote blood pressure of patients [48]. The results of our study showed that QDD could significantly inhibit ET and also has an obvious lowering effect on Ang II when being combined with antihypertensive drugs. Therefore, antihypertensive effect of QDD might attribute to inhibit the Ang II and ET release, protect the vascular endothelial cell function, and reduce the vascular remodeling. Insulin resistance was the pathophysiological basis of multiple complications in some patients with hypertension [49]. And, there was a significant correlation between insulin resistance and essential hypertension, which is one of the important causes of primary hypertension. Therefore, the current hot issue in hypertension field is how to reasonably lower blood pressure and improve insulin resistance [50]. In our systematic review, QPAD can not only increase ISI and decrease insulin resistance but also significantly reduce the INS, which provides a reliable evidence for the prevention of hypertension complications. In addition, the kidney is an organ which can expel excess metabolites through the urine and prevent proteins and blood cells from leaking out of the blood vessels. However, chronic high blood pressure may cause the protein to leak into the urine, which can damage the filter system of kidney. If the blood pressure has not been well controlled for a long time, an irreversible structural damage will be caused, which may give rise to renal impairment and even chronic renal failure. The main renal complication of hypertension is renal arteriosclerosis [51]. Therefore, the treatment of hypertension should also pay attention to the changes in renal function. In our meta-analysis, QDD showed a significant lowering effect on mA1b, *β*_2_-MG, and NAG, which proved that QDD could certainly prevent renal complications of hypertension.

**Figure 6:**
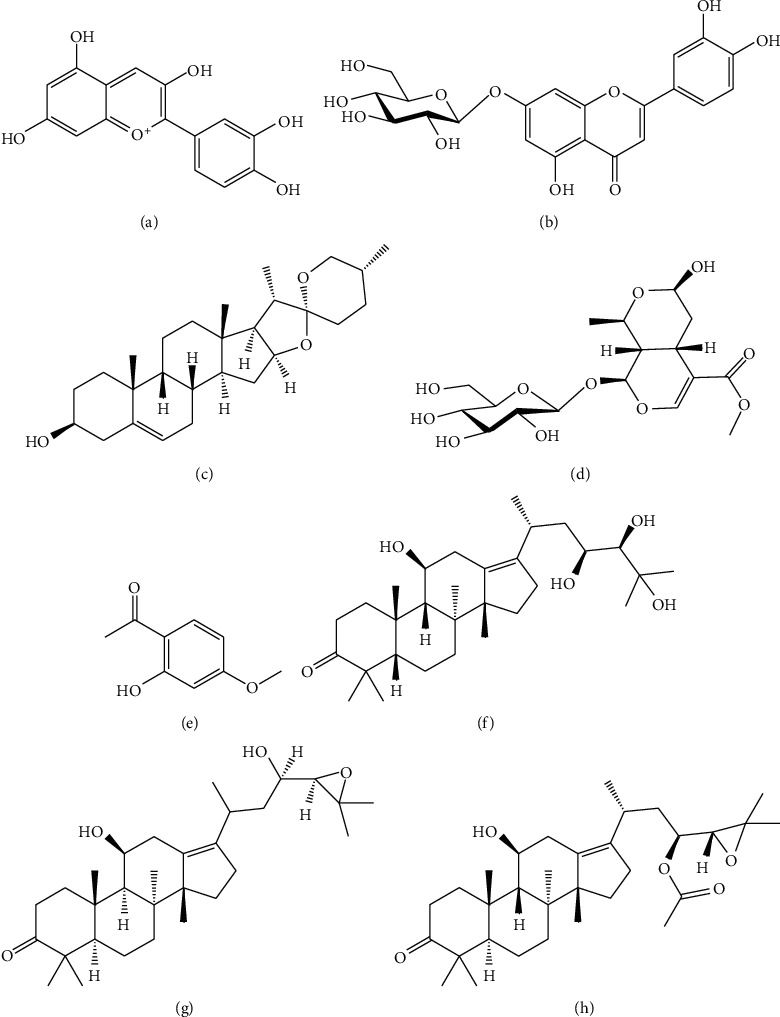
Chemical structures of the main active ingredients of QDD. (a) Anthocyanins from Gouqi, (b) cynaroside from Juhua, (c) diosgenin from Shanyao, (d) morroniside from Shanzhuyu, (e) paeonol from Mudanpi, (f) alisol A from Zexie, (g) alisol B from Zexie, and (h) alisol B 23-acetat from Zexie; abbreviations: QDD: Qiju Dihuang decoction.

QDD contains 8 single TCMs, including Lycii Fructus (Gouqizi), Chrysanthemi Flos (Juhua), Rehmanniae Radix Praeparata (Shudihuang), Dioscoreae Rhizoma (Shanyao), Corni Fructus (Shanzhuyu), Moutan Cortex (Mudanpi), Poria (Fuling), and Alismatis Rhizoma (Zexie). Anthocyanins from Gouqi (Figure 6(a)) can not only reduce blood lipid but also protect vascular endothelium, thus they may prevent atherosclerosis [52]. Total flavonoids from Juhua (Figure 6(b)) can protect the vasodilation and dilate blood vessels by controlling calcium and potassium channels, which shows that they can reduce blood pressure [53, 54]. Shudihuang has a strong effect on the cardiovascular system, and it can improve hemorheology [55]. The Shudihuang extract has a bilateral effect on blood pressure [56]. Diosgenin from Shanyao (Figure 6(c)) has obvious effects on reducing the occurrence of cardiovascular and cerebrovascular diseases because it can effectively decrease calcium overload in cardiomyocytes and regulate related signaling pathways [57]. Water extract of Shanzhuyu has an antihypertensive effect [58]. Total iridoid glycosides have a certain protective effect on the vascular endothelium of the heart and thoracic aorta, and its active component morroniside (Figure 6(d)) can prevent the injury of vascular endothelial cells by increasing the activity of SOD [59]. Paeonol from Mudanpi (Figure 6(e)) has obvious protective effect on blood vessels, which is mainly related to lowering blood lipid and inhibiting atherosclerosis. Paeonol can also significantly increase serum NO level and decrease plasma ET content [60]. Furthermore, Zexie water extract can reduce blood pressure by dilating blood vessels. Zexie terpenoids can not only inhibit the release of norepinephrine by the sympathetic nerve but also block Ca^2+^, so it can significantly lower the blood pressure [61]. Alisol A (Figure 6(f)), alisol B (Figure 6(g)), and alisol B 23-acetate (Figure 6(h)) can inhibit Ang II and reduce blood pressure [62]. In addition, the increase in blood pressure caused by the constriction of the aorta via the adrenal glands in the body can be offset by their vasodilation effect [63], and elevated blood pressure caused by the secretion of angiotensin can also be inhibited by alisol [61].

Hypertension belongs to vertigo or headache in TCM, and it often follows other symptoms according to its complex etiology. So, its treatment should consider the alleviation of some relative symptoms. Our research results showed that QPAD could significantly decrease the TCM syndrome score of patients and improve the life quality of patients. So, it has an obvious effect on reducing the clinical symptoms.

With the accelerating pace of our life, the incidence of hypertensive patients increases year by year and shows a trend of becoming younger [64]. Therefore, in addition to providing low-salt and low-fat diet, regular work, rest, and other health guidance to patients, it is particularly important to find appropriate drugs and optimized therapeutic scheme. Although antihypertensive drugs have been improved continuously in recent years, their adverse reactions cannot be avoided till now. There are various ways of treating hypertension by TCM, such as herbs, acupuncture, acupoint application, cupping, and foot bath. The multitarget of TCM exactly reflects the characteristics and advantages of integrated intervention. In recent years, many formulae have shown good antihypertensive effect [65–69]. Since hypertension has a very complicated pathological process, integrated TCM and chemical medicine treatment may be a better choice [70]. Our research results showed that QDD alone can significantly reduce the blood pressure, improve the antihypertensive effect, and inhibit the ET biological activity. Moreover, when combined with chemical medicine, QDD can also stabilize blood pressure fluctuations, decrease TCM syndrome score, inhibit inflammation, protect kidney function, reduce insulin resistance, and improve life quality of patients. Therefore, QDD combined with antihypertensive drugs is recommended to treat patients with hypertension of “liver and kidney Yin deficiency.”

Although the effects of QDD on hypertension were evaluated using a meta-analysis, there were some limitations. Firstly, though all of 19 included trials reported randomization, only 6 trials had the random number table. In 19 trials, one used a medical order and the rest of the test randomness was not clear. However, all trials had some deficiencies such as random allocation concealment, blinding of participants, personnel and outcome assessors, or selective outcome reporting. Secondly, no publication bias had been evaluated, because the number of trials included in each subgroup was too small (no more than 10). And, regarding the efficacy of QPAD for SBP, there was a greater heterogeneity among the 7 included trials. Thirdly, there are still 9 trials using modified QDD that does not exactly reflect the effect of QDD. Fourthly, QDD is widely used in the treatment of ophthalmic diseases in the clinic [8], but no outcome indicators related to ophthalmology have been reported in the included trials, so the efficacy of prevention and treatment of hypertensive ophthalmic complications remains unclear. Fifthly, the adverse reactions of QDD on hypertension are unknown, because it is not shown in the included trials.

## 5. Conclusion

In summary, this study identified that QDD can significantly reduce the blood pressure of hypertension patients and improve the antihypertensive effects; furthermore, it shows a significant ET-lowering effect. Combining with antihypertensive drugs, it can also exhibit an obvious reduction on TCM syndrome score and average blood pressure of 24 h, Ang ?, INI, mA1b, *β*_2_-MG, NAG, and HsCRP level. At the same time, QDD can improve the effect of insulin resistance and patients' life quality. However, due to some less standard design of the included randomized controlled trials, more long-term, randomized and double-blind clinical trials will be needed in the future to provide stronger evidence for the treatment of hypertension with QDD.

## Figures and Tables

**Table 1:** Characteristic of the 19 trials included in the meta-analysis.

Author(s)	Sample size (experimental/control)	Gender (male/female)	Diagnostic criteria	Experimental	Control	Duration	Outcome measures
Du et al. [20]	60/60	66/54	WHO-ISHNGMH-1999	QDD (6 g, tid) + C	Felodipine sustained release tablet (5 mg, qd)	1 month	BP, Ang II, ET
Du et al. [21]	30/30	31/29	DCPTHC	QDD (9 g, bid) + C	Nifedipine delayed-release tablet (30 mg, qd)	1 month	AE, BP
Guo and Li [22]	40/40	46/34	DCPTHC	Modified QDD (1 dose/d) + C	Nifedipine delayed-release tablet (10 mg, bid), captopril tablet (25 mg, bid)	1 month	AE, BP
Huang [23]	40/40	NR	DCPTHC	QDD (9 g, bid) + C	Nifedipine tablet (30 mg, qd)	1 month	AE, BP
Liang [24]	133/132	128/137	WHO-ISHNGMH-1999	QDD (6 g, bid) + C	CADLI	6 months	AE
Li et al. [25]	50/50	49/51	WHO-ISHNGMH-1999	Modified QDD (1 dose/d)	Conventional treatment (nao liqing, luo buma tablet, and dihydrochlorothiazide)	3 months	AE
Liu [26]	50/50	47/53	WHO-ISHNGMH-1999	Modified QDD (1 dose/d) + C	Amlodipine besylate tablet (5 mg, qd)	1 month	BP, CE, QL
Liu et al. [27]	219/114	159/174	GDTCDIMCM	Modified QDD (1 dose/d)	Conventional treatment (nao liqing, luo buma tablet, and dihydrochlorothiazide)	3 months	AE
Lu [28]	40/40	48/32	WHO-ISHNGMH-1999	Modified QDD (1 dose/d)	Captopril tablet (25 mg, tid)	NR	AE
Luo and Luo [29]	60/60	65/55	WHO-ISHNGMH-1999	Modified QDD (1 dose/d)	Nitrondipine tablet (10 mg, tid)	2 months	AE, BP
Peng et al. [30]	30/30	34/26	WHO-ISHNGMH-1999	QDD (1 dose/d)	Losartan potassium tablet (50 mg, qd)	2 months	BP, ISI, INS
Song [31]	36/36	42/30	WHO-ISHNGMH-1999	Modified QDD (1 dose/d) + C	Amlodipine besylate tablet (5 mg, qd)	1 month	BP, CE, TCMSS, QL
Sun et al. [32]	32/31	26/37	WHO-ISHNGMH-1999	QDD (1.5 g, tid) + C	Telmisartan tablet (40 mg, qd)	1 month	AE
Wang [33]	60/30	48/42	WHO-ISHNGMH-1999	Modified QDD (1 dose/d) + C	Irbesartan tablet (150 mg, qd)	2 months	BP, mA1b *β*_2_-MG, NAG
Wang et al. [34]	40/40	41/39	DCPTHC	QDD (9 g, bid) + C	Enalapril maleate and folic acid tablet (0.8 mg, qd)	1 month	AE, 24 h ABP
Yang and He [35]	40/40	46/34	DCPTHC	Modified QDD (1 dose/d) + C	Enalapril maleate capsules (5 mg, bid)	3 months	AE
Yu [36]	50/50	68/32	DCPTHC	QDD (1 dose/d) + C	Conventional antihypertensive drugs (ACEI, ARB, CCB, and diuretic)	1 month	AE, Ang II, HsCRP
Zhang [37]	34/34	30/32	WHO-ISHNGMH-1999	QDD (1 dose/d) + C	Amlodipine besylate tablet (5 mg, qd)	1 month	AE, BP
Zhu [38]	46/46	49/43	DCPTHC	QDD (9 g, bid) + C	Nifedipine controlled release tablet (30 mg, qd)	2 months	AE, BP

*Abbreviations*. ABP: ambulatory blood pressure; ACEI: angiotensin-converting enzyme inhibitors; AE: antihypertensive effect; Ang II: angiotensin II; ARB: angiotensin receptor blockers; BP: blood pressure; *β*_2_-MG: *β*_2_-microglobulin; CADLI: conventional antihypertensive drugs and lifestyle interventions; CCB: calcium channel blocker; CE: clinical effects; DCPTHC: diagnostic criteria for the prevention and treatment of hypertension in China; ET: endothelin; GDTCDIMCM: guide to diagnosis and treatment of common diseases in internal medicine of Chinese medicine; HsCRP: high-sensitivity C-reactive protein; INS: insulin; ISI: insulin sensitivity index; NAG: *N*-acetyl-*β*-glucosidase; NR: not reported; QDD: Qiju Dihuang decoction; QL: quality of life; TCMSS: TCM syndrome score; WHO-ISH GMH: World Health Organization-International Society of Hypertension Guidelines for the Management of Hypertension.

**Table 2:** The ingredients of Qiju Dihuang Decoction used in the 19 trials.

References	TCM	Ingredients of QDD
Du et al. [20]	QDD (6 g, tid)	Rehmanniae Radix Praeparata (Shudihuang), Corni Fructus (Shanzhuyu), Dioscoreae Rhizoma (Shanyao), Poria (Fuling), Moutan Cortex (Mudanpi), Alismatis Rhizoma (Zexie), Lycii Fructus (Gouqizi), Chrysanthemi Flos (Juhua)
Du et al. [21]	QDD (9 g, bid)	Rehmanniae Radix Praeparata (Shudihuang), Corni Fructus (Shanzhuyu), Dioscoreae Rhizoma (Shanyao), Poria (Fuling), Moutan Cortex (Mudanpi), Alismatis Rhizoma (Zexie), Lycii Fructus (Gouqizi), Chrysanthemi Flos (Juhua)
Guo and Li [22]	Modified QDD (1 dose/d)	Rehmanniae Radix (Shengdi) 12 g, Corni Fructus (Shanzhuyu) 9 g, Dioscoreae Rhizoma (Shanyao) 9 g, Poria (Fuling) 6 g, Moutan Cortex (Mudanpi) 6 g, Alismatis Rhizoma (Zexie) 6 g, Lycii Fructus (Gouqizi) 9 g, White Chrysanthemi Flos (Baijuhua) 12 g, Gastrodiae Rhizoma (Tianma) 9 g, Uncariae Ramulus Cum Uncis (Gouteng) 9 g, Haliotidis Concha (Shijueming) 15 g
Huang [23]	QDD (9 g, bid)	Rehmanniae Radix Praeparata (Shudihuang), Corni Fructus (Shanzhuyu), Dioscoreae Rhizoma (Shanyao), Poria (Fuling), Moutan Cortex (Mudanpi), Alismatis Rhizoma (Zexie), Lycii Fructus (Gouqizi), Chrysanthemi Flos (Juhua)
Liang [24]	QDD (6 g, bid)	Rehmanniae Radix Praeparata (Shudihuang), Corni Fructus (Shanzhuyu), Dioscoreae Rhizoma (Shanyao), Poria (Fuling), Moutan Cortex (Mudanpi), Alismatis Rhizoma (Zexie), Lycii Fructus (Gouqizi), Chrysanthemi Flos (Juhua)
Li et al. [25]	Modified QDD (1 dose/d)	Lycii Fructus (Gouqizi) 15 g, Hordei Fructus Germinatus (Shengmaiya) 15g, Chrysanthemi Flos (Juhua) 15 g, Poria (Fuling) 15 g, Rehmanniae Radix Praeparata (Shudihuang) 15 g, Alismatis Rhizoma (Zexie) 10 g, Corni Fructus (Shanzhuyu) 15 g, Dioscoreae Rhizoma (Shanyao) 15 g, Moutan Cortex (Mudanpi) 10 g, Glehniae Radix (Beishashen) 30 g, Cyathulae Radix (Chuanniuxi) 30 g, Haliotidis Concha (Shijueming) 30 g, Ostreae Concha (Shengmuli) 30 g, Raw keel (Shenglonggu) 30 g, Glycyrrhizae Radix et Rhizoma (Gancao) 6 g
Liu [26]	Modified QDD (1 dose/d)	Rehmanniae Radix Praeparata (Shudihuang) 10 g, White Chrysanthemi Flos (Baijuhua) 12 g, Lycii Fructus (Gouqizi) 15 g, Poria (Fuling) 15 g, Chinese yam (Shanyao) 15 g, Moutan Cortex (Mudanpi) 12 g, Corni Fructus (Shanzhuyu) 12 g, Alismatis Rhizoma (Zexie) 12 g, Cyathulae Radix (Niuxi) 12 g, Uncariae Ramulus Cum Uncis (Gouteng) 12 g, Gastrodiae Rhizoma (Tianma) 10 g, Magnetitum (Cishi) 30 g
Liu et al. [27]	Modified QDD (1 dose/d)	Lycii Fructus (Gouqizi) 15 g, Chrysanthemi Flos (Juhua) 15 g, Rehmanniae Radix Praeparata (Shudihuang) 15 g, Corni Fructus (Shanzhuyu) 15 g, Dioscoreae Rhizoma (Shanyao) 15 g, Radix glehniae (Beishashen) 30 g, Haliotidis Concha (Shijueming) 30 g, Raw keel (Shenglonggu) 30 g, Ostreae Concha (Shengmuli) 30 g, Cyathulae Radix (Chuanniuxi) 30 g, Moutan Cortex (Mudanpi) 10 g, Alismatis Rhizoma (Zexie) 10 g, Poria (Fuling) 15 g, Hordei Fructus Germinatus (Shengmaiya) 15 g, Glycyrrhizae Radix et Rhizoma (Gancao) 6 g
Lu [28]	Modified QDD (1 dose/d)	Lycii Fructus (Gouqizi) 25 g, Chrysanthemi Flos (Juhua) 15 g, Rehmanniae Radix Praeparata (Shudihuang) 15 g, Taxilli Herba (Sangjisheng) 15 g, Tortoise plastron (Guiban) 15 g, Cyathulae Radix (Niuxi) 15 g, Dioscoreae Rhizoma (Shanyao) 15 g, Poria (Fuling) 15 g, Corni Fructus (Shanzhuyu) 10 g, Moutan Cortex (Mudanpi) 10 g, Haliotidis Concha (Shijueming) 20 g
Luo and Luo [29]	Modified QDD (1 dose/d)	Rehmanniae Radix Praeparata (Shudihuang) 15 g, Corni Fructus (Shanzhuyu) 12 g, Dioscoreae Rhizoma (Shanyao) 15 g, Poria (Fuling) 10 g, Moutan Cortex (Mudanpi) 10 g, Alismatis Rhizoma (Zexie) 10 g, Lycii Fructus (Gouqizi) 15 g, White Chrysanthemi Flos (Baijuhua) 10 g, Ostreae Concha (Shengmuli) 15 g, Raw keel (Shenglonggu) 15 g, Haliotidis Concha (Shijueming) 10 g, Maybush (Shanzha) 15 g
Peng et al. [30]	QDD (1 dose/d)	Rehmanniae Radix Praeparata (Shudihuang), Corni Fructus (Shanzhuyu), Dioscoreae Rhizoma (Shanyao), Poria (Fuling), Moutan Cortex (Mudanpi), Alismatis Rhizoma (Zexie), Lycii Fructus (Gouqizi), Chrysanthemi Flos (Juhua)
Song [31]	Modified QDD (1 dose/d)	Rehmanniae Radix Praeparata (Shudihuang) 10 g, White Chrysanthemi Flos (Baijuhua) 12 g, Lycii Fructus (Gouqizi) 15 g, Poria (Fuling) 15 g, Dioscoreae Rhizoma (Shanyao) 15 g, Moutan Cortex (Mudanpi) 12 g, Corni Fructus (Shanzhuyu) 12 g, Alismatis Rhizoma (Zexie) 12 g, Cyathulae Radix (Niuxi) 12 g, Gastrodiae Rhizoma (Tianma) 10 g, Uncariae Ramulus Cum Uncis (Gouteng) 12 g, Magnetitum (Cishi) 30 g
Sun et al. [32]	QDD (1.5 g, tid)	Rehmanniae Radix Praeparata (Shudihuang), Corni Fructus (Shanzhuyu), Dioscoreae Rhizoma (Shanyao), Poria (Fuling), Moutan Cortex (Mudanpi), Alismatis Rhizoma (Zexie), Lycii Fructus (Gouqizi), Chrysanthemi Flos (Juhua)
Wang [33]	Modified QDD (1 dose/d)	Rehmanniae Radix Praeparata (Shudihuang) 10 g, White Chrysanthemi Flos (Baijuhua) 15 g, Lycii Fructus (Gouqizi) 15 g, Poria (Fuling) 15 g, Dioscoreae Rhizoma (Shanyao) 30 g, Moutan Cortex (Mudanpi) 15 g, Corni Fructus (Shanzhuyu) 30 g, Alismatis Rhizoma (Zexie) 15 g, Cyathulae Radix (Niuxi) 15 g, Gastrodiae Rhizoma (Tianma) 10 g, Tortoise plastron (Guiban) 15 g
Wang et al. [34]	QDD (9 g, bid)	Rehmanniae Radix Praeparata (Shudihuang), Corni Fructus (Shanzhuyu), Dioscoreae Rhizoma (Shanyao), Poria (Fuling), Moutan Cortex (Mudanpi), Alismatis Rhizoma (Zexie), Lycii Fructus (Gouqizi), Chrysanthemi Flos (Juhua)
Yang and He [35]	Modified QDD (1 dose/d)	Rehmanniae Radix (Shengdi) 12 g, Corni Fructus (Shanzhuyu) 9 g, Dioscoreae Rhizoma (Shanyao) 9 g, Poria (Fuling) 6 g, Moutan Cortex (Mudanpi) 6 g, Alismatis Rhizoma (Zexie) 6 g, Lycii Fructus (Gouqizi) 9 g, White Chrysanthemi Flos (Baijuhua) 12 g, Gastrodiae Rhizoma (Tianma) 9 g, Uncariae Ramulus Cum Uncis (Gouteng) 9 g, Haliotidis Concha (Shijueming) 15 g, Paeoniae Radix Rubra (Chishao) 9 g, Salviae Miltiorrhizae Radix et Rhizoma (Danshen) 30 g
Yu [36]	QDD (1 dose/d)	Rehmanniae Radix Praeparata (Shudihuang), Corni Fructus (Shanzhuyu), Dioscoreae Rhizoma (Shanyao), Poria (Fuling), Moutan Cortex (Mudanpi), Alismatis Rhizoma (Zexie), Lycii Fructus (Gouqizi), Chrysanthemi Flos (Juhua)
Zhang [37]	QDD (1 dose/d)	Rehmanniae Radix Praeparata (Shudihuang), Corni Fructus (Shanzhuyu), Dioscoreae Rhizoma (Shanyao), Poria (Fuling), Moutan Cortex (Mudanpi), Alismatis Rhizoma (Zexie), LyciiFructus (Gouqizi), Chrysanthemi Flos (Juhua)
Zhu [38]	QDD (9 g, bid)	Rehmanniae Radix Praeparata (Shudihuang), Corni Fructus (Shanzhuyu), Dioscoreae Rhizoma (Shanyao), Poria (Fuling), Moutan Cortex (Mudanpi), Alismatis Rhizoma (Zexie), Lycii Fructus (Gouqizi), Chrysanthemi Flos (Juhua)

*Abbreviations*. QDD: Qiju Dihuang decoction.

**Table 3:** Methodology quality of the 19 studies according to the Cochrane handbook.

References	A	B	C	D	E	F	G	H
Du et al. [20]	+	?	?	?	?	+	?	?
Du et al. [21]	?	?	?	?	?	+	?	?
Guo and Li [22]	?	?	?	?	?	+	?	?
Huang [23]	−	?	?	?	?	+	?	?
Liang [24]	?	?	?	?	?	+	?	?
Li et al. [25]	?	?	?	?	?	+	?	?
Liu [26]	?	?	?	?	?	+	?	?
Liu et al. [27]	?	?	?	?	?	+	?	?
Lu [28]	+	?	?	?	?	+	?	?
Luo and Luo [29]	+	?	?	?	?	+	?	?
Peng et al. [30]	?	?	?	?	?	+	?	?
Song [31]	?	?	?	?	?	+	?	?
Sun et al. [32]	+	?	?	?	?	+	?	?
Wang [33]	?	?	?	?	?	+	?	?
Wang et al. [34]	?	?	?	?	?	+	?	?
Yang and He [35]	+	?	?	?	?	+	?	?
Yu [36]	?	?	?	?	?	+	?	?
Zhang [37]	?	?	?	?	?	+	?	?
Zhu [38]	?	?	?	?	?	+	?	?

*Abbreviations.* A: adequate sequence generation; B: allocation concealment; C: blinding patient; D: blinding personnel; E: blinding assessor; F: incomplete outcome data; G: selective reporting; H: other bias; +: low risk; −: high risk; ?: unclear.

## Data Availability

The data used to support the findings of this study are available from the corresponding author upon request.

## Abbreviations

Ang II: Angiotensin II
*β*_2_-MG: *β*_2_-Microglobulin
CADLI: Conventional antihypertensive drugs and lifestyle interventions
CI: Confidence interval
DBP: Diastolic blood pressure
ET: Endothelin
HsCRP: High-sensitivity C-reactive protein
INS: Insulin
ISI: Insulin sensitivity index
MD: Mean difference
NAG: *N*-acetyl-*β*-glucosidase
QDD: Qiju Dihuang Decoction
QPAD: Qiju Dihuang Decoction plus antihypertensive drugs
QPCADLI: Qiju Dihuang Decoction plus conventional antihypertensive drugs and lifestyle interventions
RCTs: Randomized controlled trials
RR: Relative risk
SBP: Systolic blood pressure
TCM: Traditional Chinese medicine.
